# Melatonin free form versus its chitosan-loaded nano formula impact on wound healing of albino rat’s parotid gland “a histological study”

**DOI:** 10.1186/s12903-025-06869-4

**Published:** 2025-10-21

**Authors:** Shimaa S. Taher, Mazen Tharwat Abou Elkhier, Reham Mokhtar Aman, Essam Soussa, Samah K. Ezzat

**Affiliations:** 1 Department of Oral Biology, Faculty of Dentistry, Mansoura University, Mansoura, Egypt; 2 Department of Pharmaceutics, Faculty of Pharmacy, Mansoura University, Mansoura, Egypt

**Keywords:** Chitosan nanoparticles, Ionotropic gelation method, Melatonin, Parotid glands, Wound healing

## Abstract

**Background:**

This study was carried out in rat’s parotid glands to compare the regenerative effect of melatonin (MET) as a free drug and when loaded on chitosan nanoparticles (CNPs).

**Methods:**

Seventy-two male albino rats were divided into four groups. Circular wounds were created (5 mm diameter*2 mm depth) in right parotid glands using biopsy puncher. Wounds in group I were left to heal spontaneously while in group II received topical MET treatment. Wounds in group III received topical nanoparticles (NPs), while group IV received topical melatonin loaded chitosan nanoparticles (MET-CNPs). Rats in four groups were euthanized 3, 14, 30 days post-surgery. Salivary glands (SGs) specimens were processed for ordinary Hematoxylin and eosin (H&E) and anti-vimentin immunohistochemical (IHC) staining. Other specimens were used to demonstrate Aquaporin 5 (AQP5) expression using real time polymerase chain reaction (RT-PCR).

**Results:**

H&E findings were fibrosis, dilated, congested blood vessels, massive inflammation, and loss of the normal outline of acini with pyknosis and vacuolation after 3 days in all groups. After 14 days in group IV there were newly developed ducts and acini in the wound area. After 30 days in group IV, there was an increase in the development of ducts and acini with restoration of normal acini outline. Anti-vimentin findings were the least immunoreactivity in group IV, with means and standard deviation (SD), 1.466 ± 0.107, and highest in positive control 8.330 ± 0.214. AQP5 expression was highest after 3 days, 13.836 ± 0.004, and lowest after 14 days in group IV 8.612 ± 0.004, while in group II it was lowest after 30 days 0.256 ± 0.003.

**Conclusion:**

MET-CNPs have better healing and regenerative effects in surgical wounds of rats’ parotid glands, by enhancing the formation of new acini and ductal tissue. Also, through decreasing fibrosis as indicated by suppressed vimentin, and propagating function, as shown by increased expression of AQP5.

**Supplementary Information:**

The online version contains supplementary material available at 10.1186/s12903-025-06869-4.

## Background

Salivary glands (SGs), consist of well-differentiated epithelial cells, which have weak regenerative capabilities at sites of tissue injury, impairing their function and resulting in hypo-salivation [1]. Out of all tumors of the head and neck, salivary gland tumors (SGTs) represent around 5%. 75% arise in the parotid glands, the largest among the three sets of major SGs; 10% in the submandibular glands (SMGs); and 15% in the minor SGs of the upper digestive tract; less than 1% is present in the sublingual glands. Nearly 20% of parotid gland tumors are malignant [2].

Parotid salivary glands show similarities between humans and rats. However, rats’ parotid is the second-largest major salivary gland, while in humans they are the Largest. Additionally, in rats, the intercalated duct epithelium is low cuboidal and joins directly to intralobular ducts, while in humans intercalated duct epithelium is cuboidal; intralobular ducts are larger in serous glands than in mucous. The duct opens near the mandibular molars in rats. While Stensen’s duct opens in the mouth by the second maxillary molar in humans [3].

Vimentin, a type III cytoplasmic intermediate filament protein, is specific to mesenchymal and endothelial cells. It is involved in wound healing, where the function of vimentin is coordinating fibroblast proliferation and keratinocyte differentiation during wound healing. Loss of vimentin led to a severe deficiency in fibroblast growth, which inhibited the activation of two major initiators of epithelial–mesenchymal transition (EMT), TGF-β1 [4, 5].

Aquaporins (AQPs) are proteins that can selectively control water transport across membranes. Aquaporin 5 (AQP5) is a widely used marker to assess the drying effect of chemicals or degenerative changes on salivary glands. The decline in AQP5 secretion may be attributed to increased free radicals and oxidative stress, and the morphometric changes in the salivary glands [6].

Melatonin (MET) is an indolamine produced primarily by the pineal gland, and other tissues, as the retina, gut, and skin. MET is considered an antioxidant owing to its potential in neutralizing the generation of free radicals and reactive oxygen species (ROS) [7]. It affects the activities of keratinocytes and has anti-inflammatory and neuroprotective actions. Several in vivo studies demonstrated that MET has an ameliorative effect on wound healing. It has been utilized in multiple preparations for topical application, both free and encapsulated in nanocarriers [8, 9].

Chitosan, an active and deacetylated chitin derivative, has been utilized in healing medicines, as it provides numerous features which could enhance this process. It acts positively in hemostasis and tissue regeneration, triggers the formation of fibroblasts and has antifungal and antimicrobial actions [10, 11]. Additionally, it is a biocompatible and biodegradable polymer demonstrating mucoadhesive characteristics. The polyanions as sodium tripolyphosphate (TPP) and Apple pectin (AP) are usually used as a cross-linking agents with chitosan [12].

Nanotechnology has gained much more popularity in recent years, as it can solve particular issues accompanied by conventional administrative routes. Nanoparticles (NPs) could be optimized to improve not only drug efficacy, but also their safety, by controlling their amount, time and release in the site of action, and their bioavailability, either by increasing absorption through enhanced solubility or by facilitating their passage through the biological membranes [13].

This study aimed to enhance the regenerative potential of SG cells instead of scar tissue formation. This is via increasing the infiltration of fibroblasts from the nearby connective tissue into the wound region and accelerating the differentiation of progenitor cells. The null hypothesis was that there is no significant difference between MET in its free form and when loaded on chitosan NPs.

## Methods

### Sample size and animal subjects

The sample size was determined using G*Power version 3.1.9.4. The calculated sample size was 72 adult male albino rats, weighting 180 to 200 gm. All procedures of the experiment are reported in accordance with Animal Research: Reporting of In Vivo Experiments (ARRIVE) guidelines for the reporting of animal experiments (httpt://www.arriveguidelines.org), and the Declaration of Helsinki [14].

### Study design

Rats were randomly divided (using random number tables) into 4 groups (*n* = 18).

**Fig. 1:**
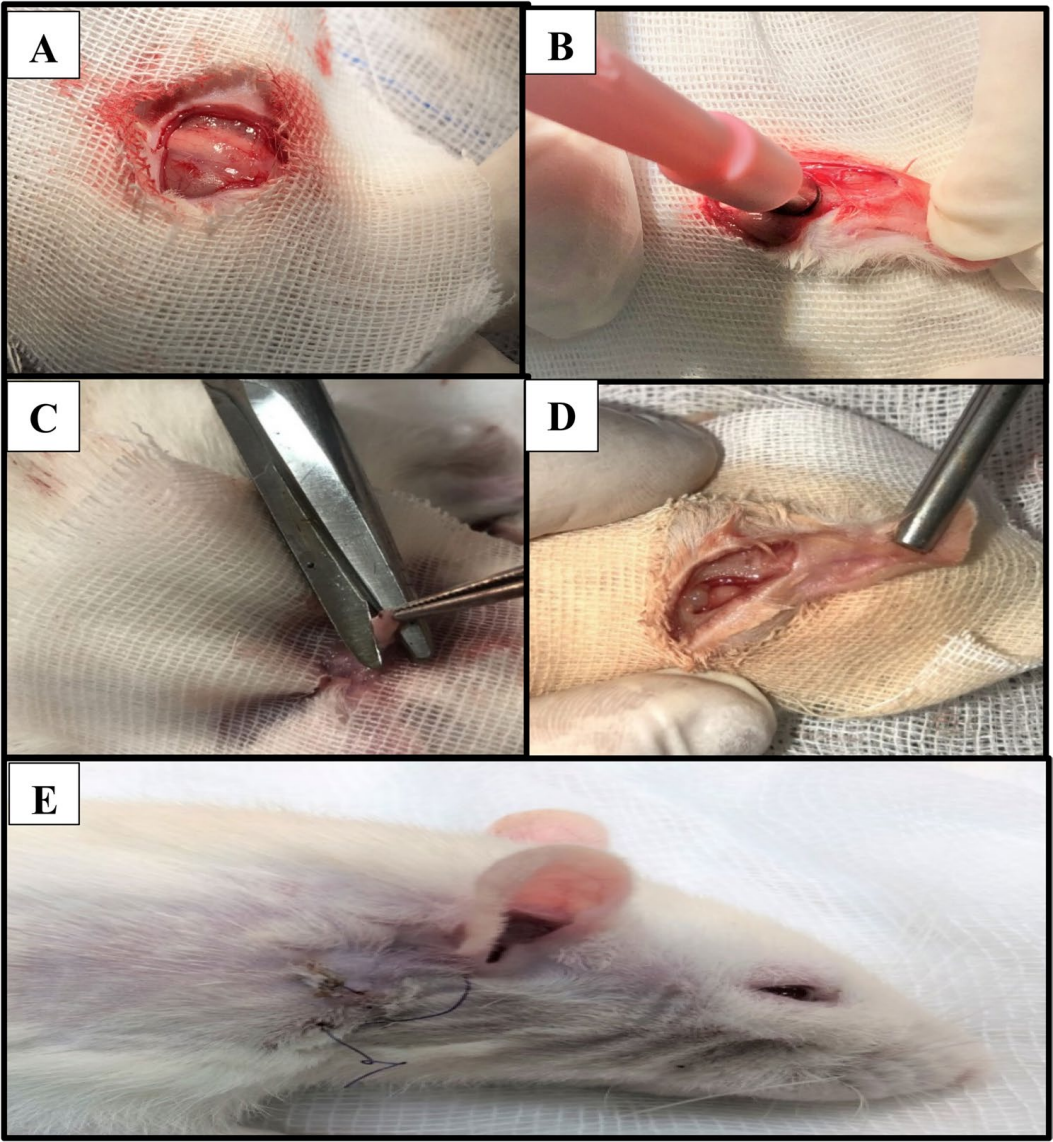
shows surgical procedures: **A** Skin incision, **B&C** Wound creation, **D **Created wound, **E** Wound closure
- Group II (MET group): surgical wounds were performed as in group I, then treated with pure MET (2 mg/rat MET suspended in 1% w/v sodium carboxymethylcellulose (sodium CMC)) [15].
- Group III (NPs group): rats were handled surgically just like previous groups, then treated with the nanocarrier (plain chitosan nanoparticles (P CNPs) corresponding to the medicated formula).
- Group IV (melatonin-loaded chitosan nanoparticles (MET-CNPs) group): rats were handled surgically just as in previous groups, then treated with the MET-CNPs formula (2 mg/rat) [15].

- Group I (positive control group): right parotid glands were approached extraorally, then circular wounds by a biopsy puncher with 5 mm diameter * 2 mm depth on the upper portion of the gland were made. Rats were left without treatment except saline irrigation before suturing **(**Fig. 1**)**.

MET dose was selected for the experiment based on the results of a previous study regarding topical application of MET loaded lecithin-chitosan NPs to improve the wound healing in diabetic rats [15]. Animals were anesthetized with intra-peritoneal injection of ketamine hydrochloride^®^ by dose 75 mg/kg & Xyla-Ject^®^ by dose 5–10 mg/kg. Six rats from each group were euthanized on after operation day (AOD) 3, 14 and 30 by overdose of halothane.

### Surgical procedures

All surgical steps were performed in Mansoura Experimental Research Center (MERC).

### Drug preparation

#### Chemicals

Chitosan (Degree of deacetylation: 75–85%, Molecular weight: 50–190 KDa, CAS Number: 9012-76-4, Product Number: 448869), TPP and MET were purchased from Sigma-Aldrich (Saint Louis, MO 63103, USA). AP (CAS Number: 9000-69-5, Lot No.: 2IE0212) was kindly provided by Spectrum Chemical Mfg. Corp. (New Brunswick, NJ, USA). Analytical grade of glacial acetic acid (99%) and sodium CMC were procured from El-Nasr Pharmaceutical Chemical Co. (Cairo, Egypt).

#### Methods

##### **Preparation of MET-CNPs**

MET-CNPs were prepared following the ionotropic gelation method that depends on the ionic interaction between the positively-charged chitosan (cationic part) and the negatively-charged mixture of TPP-AP blended solution (anionic part) (16).

Succinctly, utilizing an ultrasonic bath (Sonix IV, SS101H 230, ETL Testing Laboratories Inc., USA), an accurate weight of total MET (40 mg) was dissolved in aqueous acetic acid solution (25 mL, 0.5% v/v) for 3 h. Subsequently, to acquire a final concentration of 0.25% w/v, the calculated chitosan (cationic part) quantity was weighed accurately, added to the above-prepared MET aqueous acetic acid solution, magnetically stirred using magnetic stirrers (MS300HS, MTOPS Corp., Korea) until complete dissolution and filtered finally via membrane filters (0.45 µm, EMD Milli- pore, Billerica, MA, USA). Concurrently, to prepare the crosslinking agent (anionic part), both TPP and AP were separately dissolved in distilled water, filtered, and blended at a w/w ratio of 1:2.

At room temperature, the MET-CNPs were formed spontaneously by the drop wise addition of TPP-AP blended solution, utilizing a disposable insulin needle, on chitosan aqueous acetic acid solution containing MET, to obtain a final w/w ratio between chitosan and TPP-AP blend of 1:1. The crosslinking reaction occurred under the following circumstances: (Distance between needle tip and the surface of chitosan solution: 7 cm, the dropping rate: 0.33 mL/min, stirring speed: 1200 rpm). Moreover, unceasing stirring for an additional 30 min was adopted to permit further crosslinking reaction to continue. Similarly, P CNPs were prepared, without the incorporation of MET in the chitosan aqueous acetic acid solution. 

After all, utilizing a cooling centrifuge (ACCULAB, CE16-4X100RD, and USA) at 13,000 rpm for 1.5 h at 4 °C, the developed MET-CNPs besides its corresponding P CNPs were centrifuged to separate the clear supernatant from the precipitated NPs. The MET-CNPs'assembled supernatant would be kept for estimation of percent entrapment efficiency (EE %), using P CNPs'supernatant as a blank.

##### **Evaluation of MET-CNPs**

Three identical samples of freshly-made MET-CNPs preparations were analyzed for their average particle size (Z-average), particle size distribution (polydispersity index, PDI), and zeta potential (ζ-potential), after proper dilution with ultrapure water (UPW), utilizing a Zetasizer Nano ZS90 (Malvern Instruments, Malvern, UK). The obtained values were presented as mean ± standard deviation (SD).

After cooling centrifugation, EE % of the developed MET-CNPs, in triplicates, were determined indirectly (16). Free MET was detected in the MET-CNPs'gathered supernatant by ultraviolet/visible (UV–VIS) spectrophotometer at 278 nm (JENWAY 6850, UV–VIS double beam spectrophotometer, UK), versus P CNPs'supernatant as a blank. EE % was calculated as follows “Equation (1)”:

1 $$EE\%=\left(\mathrm{Total}\;\mathrm{MET}-\mathrm{Free}\;\mathrm{MET}\right)/\mathrm{Total}\;\mathrm{MET}\times100$$

The morphology of MET-CNPs was observed by transmission electron microscopy (TEM, JEOL, JEM-2100, JEOL Ltd., Tokyo, Japan). Briefly, a sample of NPs colloidal dispersion was diluted with UPW, a drop of such diluted sample was deposited over a carbon-coated copper grid and air-dried at room temperature. Thereupon and without staining, the sample was examined under TEM equipped with a high-resolution digital camera. 

### Evaluation methods

#### Histological evaluation

The parotid glands were fixed immediately in 10% formaldehyde prepared in phosphate buffer saline (PBS) for 24 h. after that tissue processing was completed using an automatic tissue processor. Hematoxylin and eosin (H&E) staining procedures were performed according to the protocol of Fischer et al. [17].

#### Immunohistochemical (IHC) evaluation

IHC staining was used for evaluating the expression of vimentin: Anti-vimentin (GenomeMe, Richmond, Canada). IHC slides were photographed using Olympus^®^ digital camera installed on Olympus^®^ microscope with 1/2 X photo adaptor, using 40X objective. Every section was evaluated blindly by two histopathologists. The result images were analyzed on Intel^®^ Core I7^®^ based computer using VideoTest Morphology^®^ software (Russia) with a specific built-in routine for area, % area, measurement, object counting and contact Angle.

##### **Statistical analysis**

Data were entered and analyzed by using IBM-SPSS software (version 27, 2020), and JAMOVI Desktop Software (version 2.4.8). A two-way Analysis of Variance (ANOVA) was used to determine whether there is an interaction effect between two independent categorical variables (group and time) on a continuous dependent variable.

#### Real time polymerase chain reaction (RT-PCR)

The parotid SGs were preserved in liquid nitrogen to be utilized for extraction and isolation of RNA to quantify AQP5 via RT-PCR. All steps were carried out following the manufacturing instructions [18]. (TRIzol^®^ Reagent (Invitrogen, Carlsbad, CA, USA), (QuantiTect Reverse Transcription Kit* (Qiagen, Germantown, MD, USA). (HERAPLUS SYBR^®^ Green qPCR (Willowfort, Birmingham, UK), (Real-time PCR 7500 fast system* (Applied Biosystems; Life Technologies, Carlsbad, CA, USA).

## Results

### Preparation and characterization of MET-CNPs

**Fig. 2:**
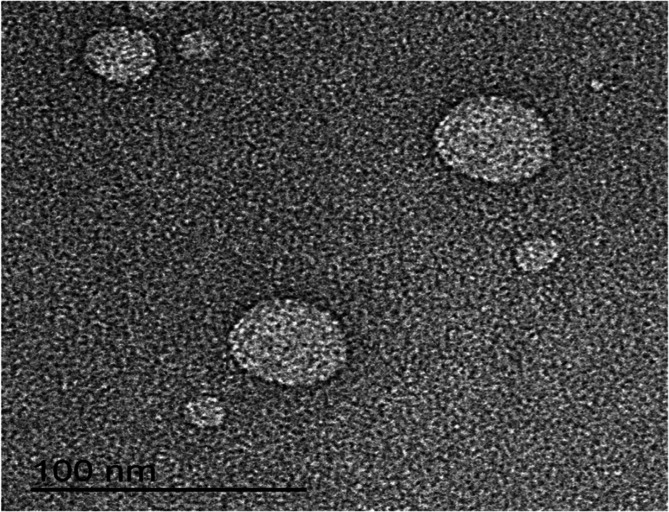
TEM image of MET-CNPs

The used w/w ratio of TPP-AP blend as well as that between chitosan and TPP-AP blend was chosen based on circumstantial physicochemical studies (data not shown). Concisely, different w/w ratios of TPP-AP blends besides those between chitosan and TPP-AP blends were used and evaluated. The most suitable w/w ratios were TPP and AP blended solution at 1:2 ratio, besides that between chitosan and TPP-AP blend of 1:1 ratio. The developed MET-CNPs exhibited Z-average, PDI ζ-potential, and EE % values of 238.03 ± 23.25 nm, 0.47 ± 0.02, + 27.90 ± 1.23 mV, and 56.69 ± 1.56%, respectively. Microscopical imaging of the investigated MET-CNPs dispersions revealed a spherical shape with smooth surface. Discernibly, Z-average of the scrutinized CNPs assessed by the Zetasizer (238.03 nm) exceeded that evaluated by TEM (˂100 nm) **(**Fig. 2**)**.

### H&E results

**Fig. 3:**
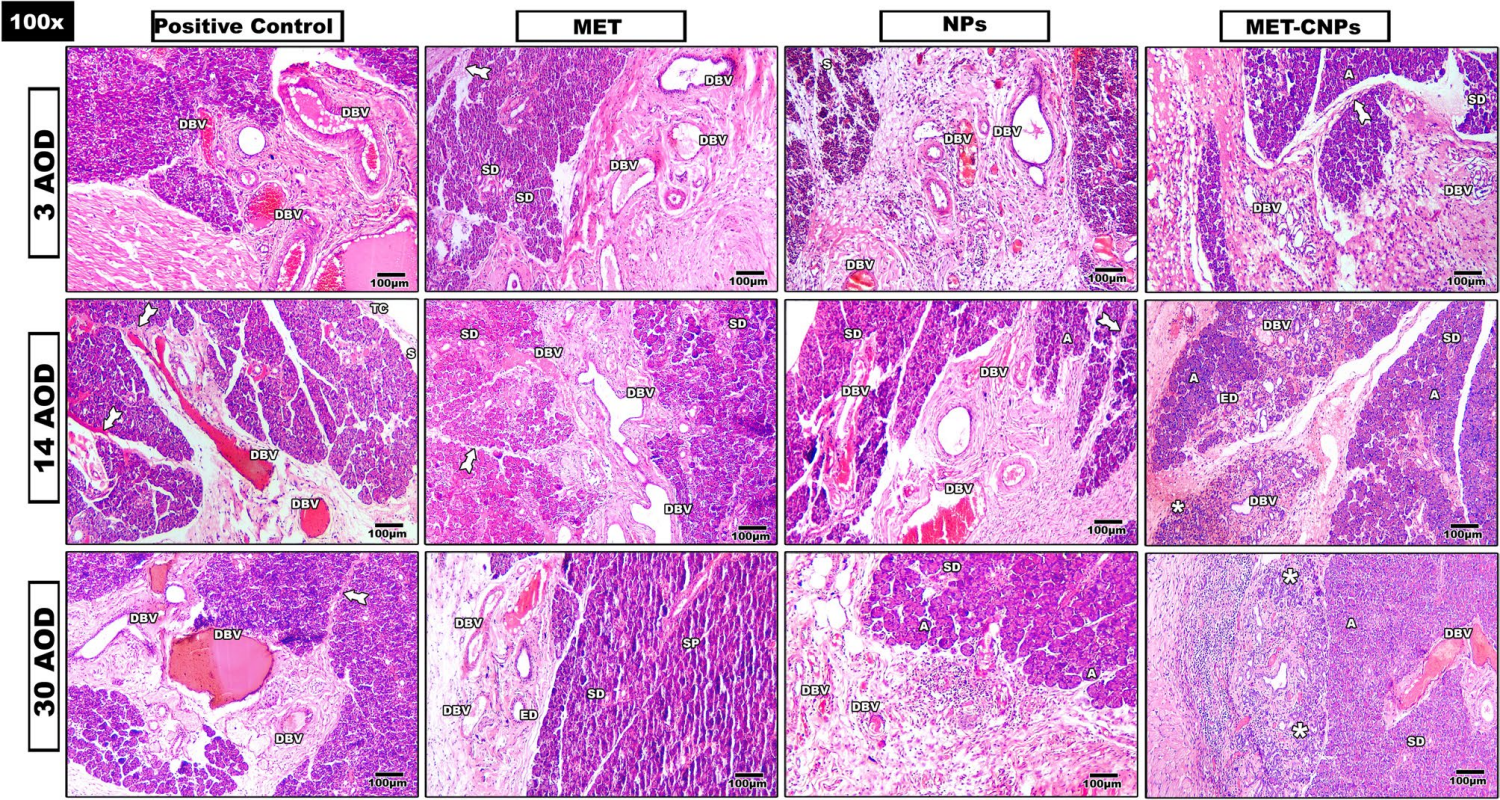
Composite photomicrograph of all studied groups stained with H&E. DBV: dilated blood vessel, SD: striated duct, A: Acini, S: separation, ED: Excretory duct, TC: Thick capsule, Tailed arrow: Thick Interlobular connective tissue septa, SP: Septa, asterisk: Newly developed ducts and acini (X100)

**Fig. 4:**
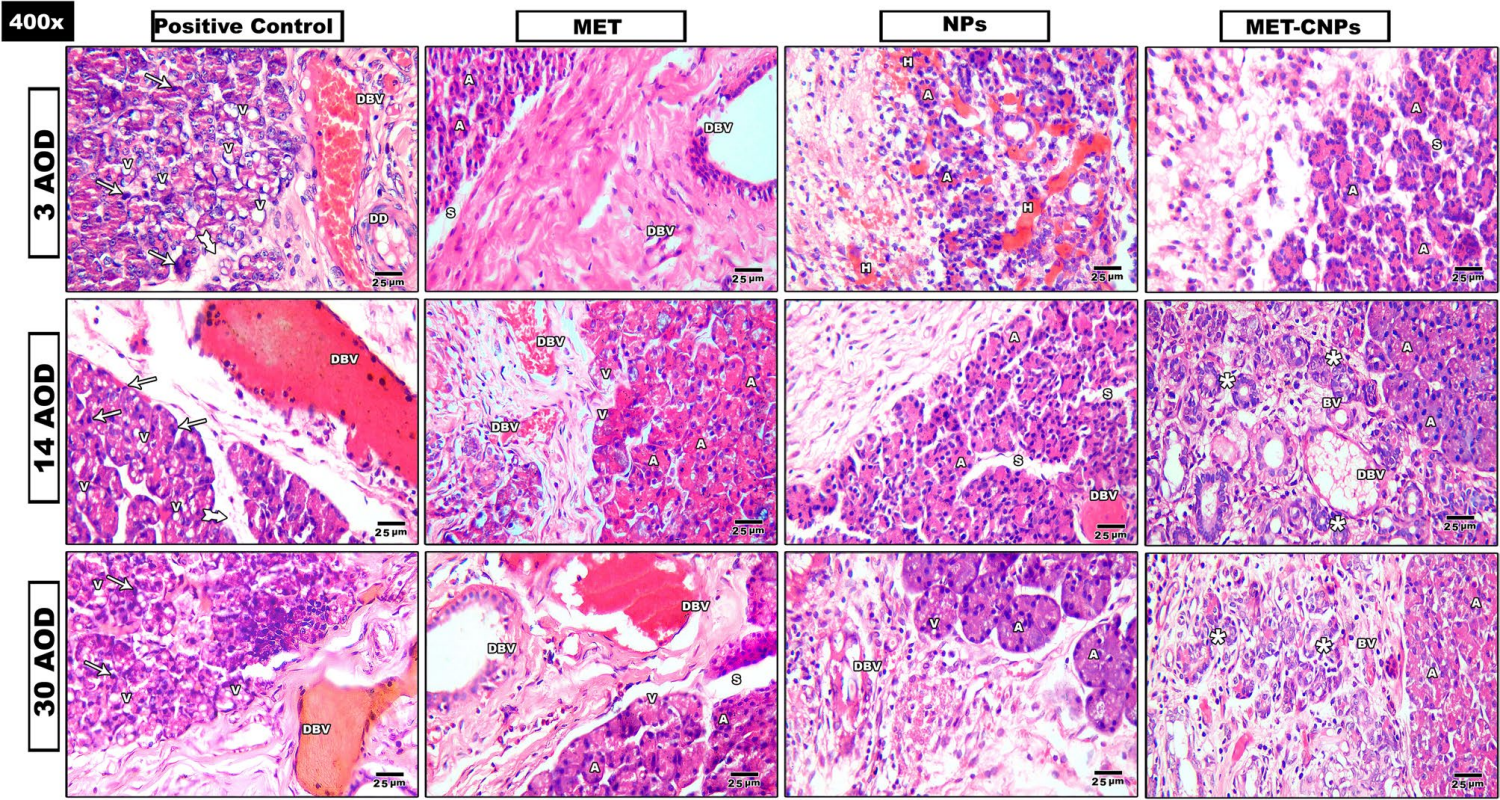
Composite photomicrograph of all studied groups stained with H&E. DBV: dilated blood vessel, DD: Dilated duct, A: Acini, S: separation, V: vacuolation, Tailed arrow: Thick Interlobular connective tissue septa, H: Hemorrhage, asterisk: Newly developed ducts and acini, BV: Blood vessel (X400)

Microscopic examination of the H&E stained sections of the studied groups **(**Figs. 3 and 4**)** revealed that:

#### Positive control group

Three days AOD, the wound area was filled with fibrous tissue. Multiple dilated blood vessels were observed. Changes in the outline of the acini were observed. Fourteen days AOD, the underlying serous acini appeared with loss of their connection. Many dilated ducts with hemorrhagic areas were also noticed. Thirty days AOD, the wound was filled with granulation tissue. Vacuolation and pyknosis were seen in acinar and ductal cells.

#### MET group

Three days AOD, the wound area was filled dense inflammatory cells infiltration. Increased thickness of interlobular connective tissue septa was also seen. Fourteen days AOD, the wound area was filled with extravasated RBCs with some decrease in blood vessels wall thickness. Thirty days AOD, noticeable loss of adherence between the acini was seen in underlying glandular tissue.

#### NPs group

Three days AOD, the wound area was seen with massive inflammation, a lot of fibrous tissue with areas of hemorrhage. Fourteen days AOD, some of the underlining acini were seen with slight restoration of its normal architecture and loss of adherence between them. Thirty days AOD, the wound area was found with fibrosis and some decrease in vascularity.

#### MET-CNPs group

Three days AOD, the area of wound was filled with fibrous tissue, inflammatory cells and the detected ducts were found with normal outline. Fourteen days AOD, newly formed ducts and acini were detected in wound area. Thirteen days AOD, in the wound area, increase in the newly formed ducts and acini were detected when compared with 14 days AOD. In the undelaying glandular tissue, the duct system was seen with normal histology.

### IHC findings

#### Anti-vimentin (Fig. 5)

By the measurement of area percent of vimentin positive immunoexpression:

**Fig. 5:**
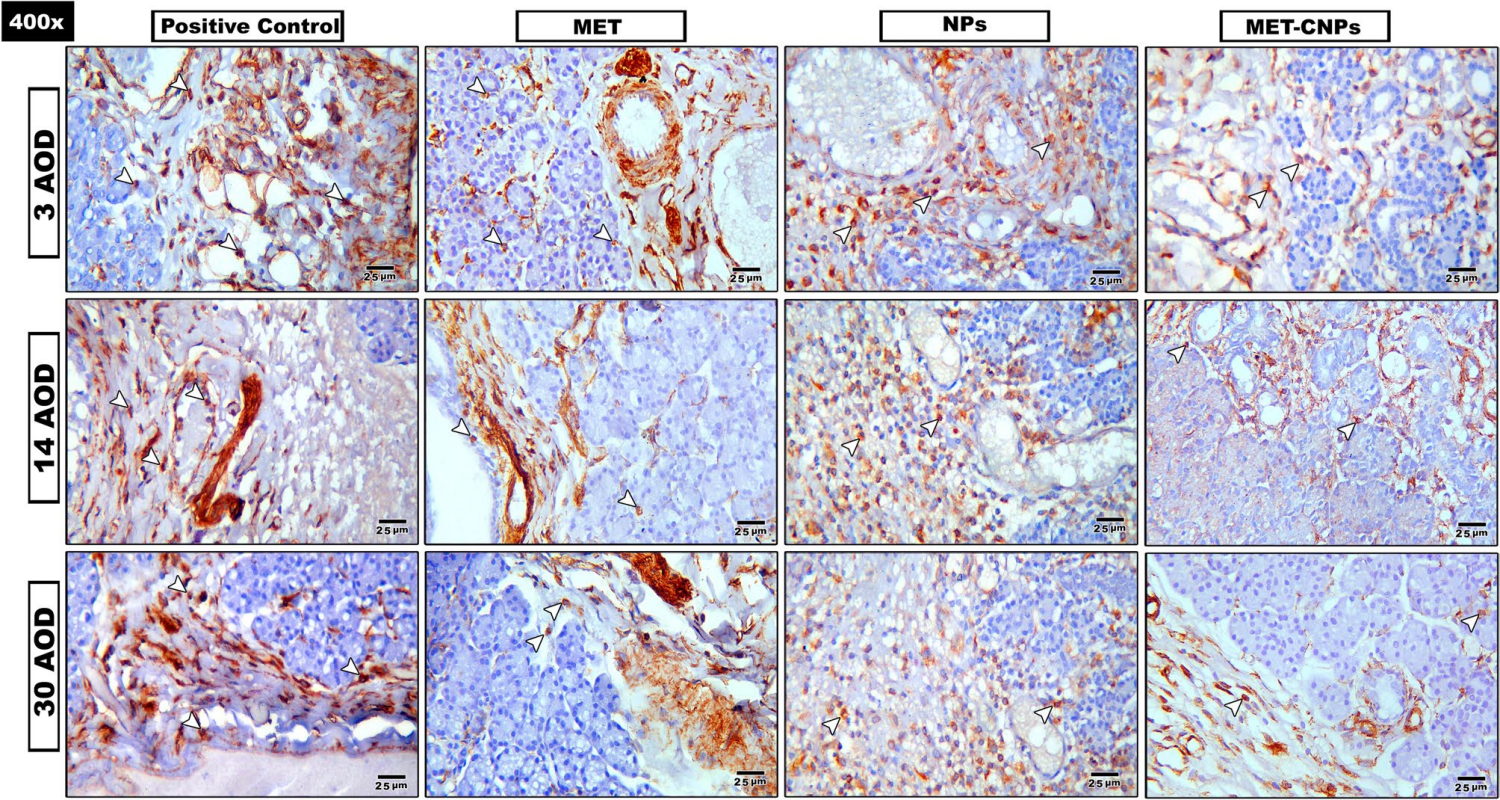
Composite photomicrograph of all studied groups stained with anti-vimentin IHC stain, Arrow Head: Brown discoloration of positive immunoreactivity (X400)

##### **Positive control group**

Three days AOD: showed intense +ve immunoreactivity (11.4%) appeared throughout the gland. Fourteen days AOD: showed moderate +ve immunoreactivity (6.7%) appeared throughout the gland. Thirty days AOD: showed intense +ve immunoreactivity (8.4%) appeared throughout the gland.

##### **MET group**

Three days AOD: showed moderate +ve immunoreactivity (6.7%) appeared throughout the gland.Fourteen days AOD: showed moderate +ve immunoreactivity (4.8%) appeared throughout the gland.Thirty days AOD: showed weak +ve immunoreactivity (3.3%) appeared throughout the gland.

##### **NPs group**

Three days AOD: showed moderate +ve immunoreactivity (4.9%) appeared throughout the gland. Fourteen days AOD: showed weak +ve immunoreactivity (3.7%) appeared throughout the gland. Thirty days AOD: showed weak +ve immunoreactivity (2.6%) appeared throughout the gland.

##### **MET-CNPs**

Three days AOD: showed weak +ve immunoreactivity (2.4%) appeared throughout the gland. Fourteen days AOD: showed weak +ve immunoreactivity (1.8%) appeared throughout the gland. Thirty days AOD: showed weak +ve immunoreactivity (1.5%) appeared throughout the gland.

### Statistical analysis

#### Anti-vimentin

**Fig. 6:**
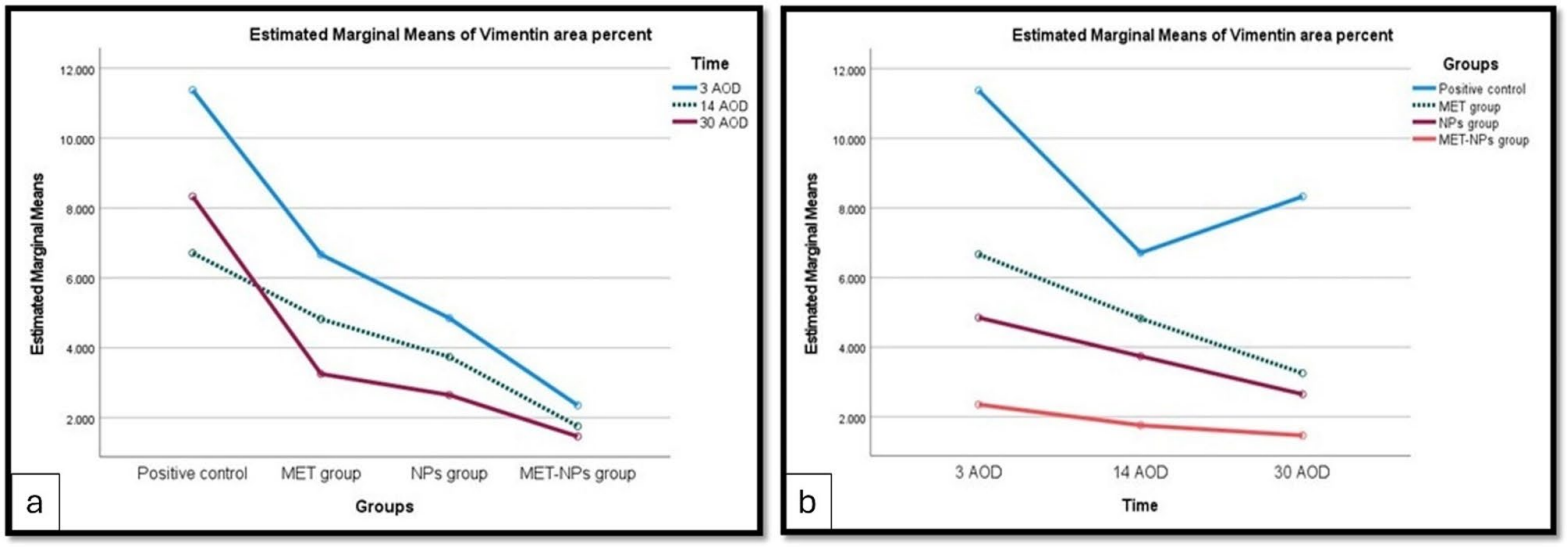
Mean Vimentin expression in 4 groups at each period (**a**). Mean expression of Vimentin in 3 time periods in each group (**b**)

A two-way ANOVA was conducted to examine the effects of group and time on Vimentin. There was a statistically significant interaction between group and time, F (6, 60) = 339.557, *p <* 0.001, partial η^2^ = 0.971 **(**Table 1; Fig. 6a, b**).** In all three treatment groups, Vimentin was statistically significantly lower in 30 AOD < 14 AOD < 3 AOD, except for 14 AOD vs. 30 AOD in MET-CNPs group.

**Table 1:** Descriptive statistics of vimentin in the 4 groups at the 3 time periods

	Mean ± SD for *n* = 6		
Time	3 AOD	14 AOD	30 AOD
Positive control	11.375 ± 0.166	6.713 ± 0.144	8.330 ± 0.214
MET group	6.670 ± 0.142^a^	4.825 ± 0.142^a^	3.254 ± 0.134^a^
NPs group	4.851 ± 0.121^a^	3.741 ± 0.119^a^	2.651 ± 0.117^a^
MET-CNPs group	2.355 ± 0.111^a^	1.758 ± 0.108^a^	1.466 ± 0.107^a^

^a^ Statistically significant at *p <* 0.001

*SD* Standard deviation

#### AQP5

**Fig. 7:**
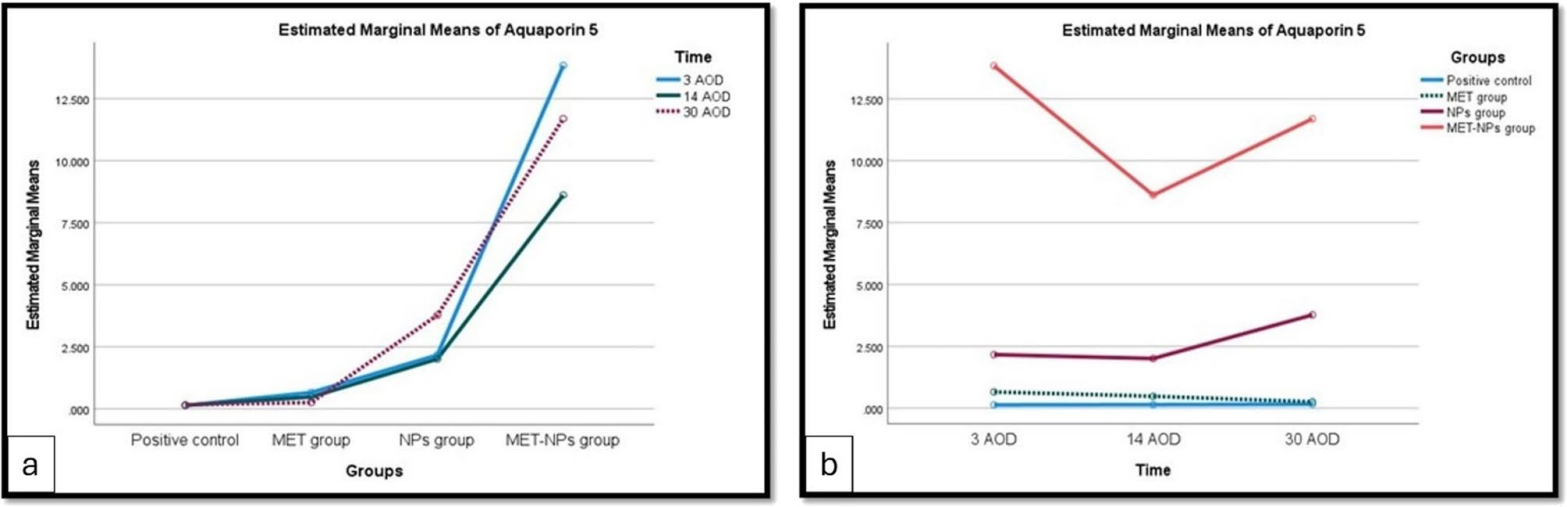
Mean AQP5 expression in 4 groups at each period (a). Mean expression of AQP5 in 3 periods in each group (b)

A two-way ANOVA was conducted to examine the effects of group and time on AQP5. There was a statistically significant interaction between groups and time on AQP5, F (6, 60) = 10250580.5, *p <* 0.001, partial η^2^ = 1.0 **(**Table 2 and Fig. 7a, b**).** AQP5 was statistically significantly higher in MET-CNPs group < NPs group < MET group < positive control, at 3 AOD, 14 AOD, and 30 AOD.

**Table 2:** Descriptive statistics of AQP5 in the 4 groups at the 3 time periods

	Mean ± SD for *n* = 6		
Time	3 AOD	14 AOD	30 AOD
Positive control	0.133 ± 0.003	0.142 ± 0.001	0.162 ± 0.002
MET group	0.657 ± 0.004^a^	0.480 ± 0.002^a^	0.256 ± 0.003
NPs group	2.163 ± 0.003^a^	2.006 ± 0.004^a^	3.770 ± 0.004^a^
MET-CNPs group	13.836 ± 0.004^a^	8.612 ± 0.004^a^	11.691 ± 0.003^a^

*SD* Standard deviation

^a^ Statistically significant at *p <* 0.001

## Discussion

In the present study, MET-CNPs were successfully prepared by ionotropic gelation method, where negatively-charged mixture of TPP-AP blended solution interacted with the positively-charged chitosan through crosslinking reaction. This method is a simple and low-cost process. Additionally, chitosan is recognized to be the most versatile biopolymer among the polymer classes amenable of ionic crosslinking [19]. Besides, this polymer has been ratified by the Food and Drug Administration (FDA) as “Generally Recognized as Safe” (GRAS) and is approved for use in drug delivery applications [20].

Regarding nanoparticulate drug delivery systems (DDs), both Z-average and PDI are the parameters that have been most commonly linked to NPs’ bioavailability, besides their biodistribution. It could be inferred that the small Z-average of MET-CNPs might be ascribed to higher AP ratio, in the anionic mixture of TPP-AP blended solution, as an optimum AP amount promoted the formation of more dense and rigid polymeric matrix with diminished Z-average [16]. Moreover, the small PDI value (< 0.5) depicts narrow Z-average distribution and is referred to as monodispersed colloidal particles.

The attained positively-charged ζ-potential value, that can be attributed to the presence of freely ionized amino groups (–NH3^+^) on the surface of the CNPs, favors adhesion to the negatively-charged cell mucosa, thence can be used as mucoadhesive DDs [16, 21]. The reasonable EE % value of 56.69 ± 1.56% indicated an efficient ionic crosslinking reaction between chitosan and TPP-AP blend, besides, an optimum AP amount promoted the formation of more compact and rigid polymeric network. As a result, the enhanced crosslinking effect of the TPP-AP anionic mixture as well as the more compact structure facilitated MET encapsulation [16].

Furthermore, Z-average’ diminution of the MET-CNPs when displayed by TEM, compared to that estimated by Zetasizer, is supposed to be attributed to different mechanisms of both measurements. Where, Zetasizer measures the hydrodynamic diameter of the NPs aqueous colloidal dispersion, whilst TEM measures the actual diameter of the dried NPs, as reported previously [21–23]. Substantially, the achieved results clarified the successful preparation of a monodispersed colloidal nanoparticulate system that could load MET efficiently.

In the present study, the selection of surgical wound creation in parotid glands depended on that SGTs represent 3–10% of the total. The parotid glands is the source of about 80% of these tumors, and 80% of them are benign. In SGTs, surgical intervention is considered an effective local treatment either alone or in combination with other therapeutic modalities [24]. This study was designed to compare the healing and regenerative capacity of MET as a free drug or loaded in chitosan, as a nanocarrier, in surgical wound created in rat parotid glands.

Acinar atrophy, which was reported in our study, was explained by Alghonemy WY et al. (2023), who studied the impact of bone marrow mesenchymal stem cells in the regeneration of submandibular salivary gland in Albino rat, suggested that gland atrophy is caused by parasympathetic nerve damage and severe irreversible atrophic changes in gland structure. Denervation, adrenergic antagonists, duct ligation, or liquid diets [25] may also contribute to acinar atrophy.

Cytoplasmic vacuolization demonstrated in the serous acinar cells as well as in the duct cells in the present study could be explained according to Shubin et al. (2016), who referred to it as the reduction in the water content in the cytoplasm of apoptotic cells. Subsequently, the cells compensate for this by swelling and vacuolization. Furthermore, they reported that intense vacuolization that could be lipid-filled or autophagic vacuoles led to cell death, either of the lytic or apoptotic type [26].

Duct dilatation occurred whenever the gland was atrophied or there was impairment in its function. In addition to vacuolization and ductal dilatation, the presence of congested blood vessels was evident. Hishida S et al. (2016) reported an increased vascularity in duct-ligated rat SMGs [27].

In the MET group, 30 days AOD, parenchymal tissues showed some restoration of normal acinar and ductal configuration. These results can be interpreted to indicate that MET induces protein synthesis in the rat parotid gland and thereby affects glandular activity in a MET1 and MET2 receptor-mediated mechanism [28]. These results agreed with our previous study [29] and in consolidation with Aras S et al. (2022), Elsherbini AM & Ezzat SK (2020), and Cakmak Karaer I et al. (2016) as they studied the MET’s protective effect on SGs tissue. Also, when MET was compared with other materials, such as royal jelly, it revealed better wound healing potential in geriatric and young mice [30–33].

NPs group wounds were treated CNPs. This group findings in agreement with Kobayashi F et al. (2019), as they studied the effect of fibroblast growth factor 7 (FGF7) during wound healing of rat submandibular gland [34]. Also Matsuzaka K et al. (2016), developed an animal model of surgically wounded SMGs and investigated the effects of collagen gel with basic fibroblast growth factor (bFGF) on tissue regeneration of surgically wounded SMGs in vivo [35]. Moreover, Meabed O.M et al. (2023) proved that CNPs enhance salivary gland branching and regulate the development of progenitor cells via increasing basement membrane (BM) components expression, such as collagen, laminin, and heparan sulphate proteoglycan [36].

In the present study, Group IV was treated with MET-CNPs. Findings revealed a different and better impact on the glandular tissues in comparison with other groups at the same duration period, and this could be attributed to loading MET in CNPs. As MET has a short half-life, prolonged release of MET has shown greater efficacy and safety when combined with a nanostructured formulation [37].

In MET-CNPs 14 and 30 days AOD, newly formed ducts and serous acini have been detected in the wound areas with a slight increase in the amount at 30 days when compared with 14 days. In the surrounding parenchymal tissues, there was restoration of normal histology and outline of the ducts and acini.

According to Sohn EH et al. (2024), MET, when administered topically or systemically, stimulates fibroblasts to produce collagen fibers, growth factors, and modulate MMP activity that organizes the wound healing process and cell proliferation [38].

Liu Y et al. (2024) reported in their research that MET prevented glandular damage and preserved function in SGs of Primary Sjogren’s syndrome, by potentially suppressing inflammation, oxidative stress, and apoptosis by inhibiting the IL-6/STAT3 pathway through receptor-dependent mechanisms [39].

By encapsulating MET within NPs, its solubility and stability can be increased, as well as the duration of its pharmacological effects, where MET in its pure form has a short half-life; so prolonged release of MET has shown greater efficacy and safety. Formulations containing NPs were found to potentiate the beginning of the proliferative phase via increasing fibroblast proliferation and blood vessel count, improving the healing process [15].

In the present study, statistical analysis of % area of vimentin reaction was performed, and there was a significant interaction between groups and times. At the group level, vimentin expression at all-time durations was lowest in MET-CNPs group and highest in the control positive group. At the level of time durations, vimentin expression in all treated groups was highest after 3 days and lowest after 30 days.

A study conducted by Luitje ME et al. (2021), they investigated human SMGs and parotid glands after radiation treatment for head and neck cancers and they found that treated samples showed a significantly higher level of vimentin staining. They explained that these results were associated with a reduced number of surviving or recovered acinar cells, and increased fibrosis [40]. These results are in agreement with our results in relation to fibrosis.

In the present study, the mRNA expression of AQP5 has been evaluated. As regarding to the different groups, it was higher in MET-CNPs than MET group. In control positive group as a model of healing without treatment, its expression is time dependent and increase gradually from day 3 to be the highest after 30 days.

In MET group, its expression decrease gradually by time to be the lowest after 30 days. In MET-CNPs group, its expression was the highest after 3 days then decreased after 14 days and finally increased again after 30 days. In group treated with NPs only, its expression was lowest after 14 days then increased again to be highest after 30 days.

A study performed by Yasumitsu T et al. (2018), in their study they evaluated the localization of AQP5 during regeneration of rat SMGs after duct ligation. After duct ligation for 7 days, the regenerating glands were collected on days 0, 1, 3, 7, and 14 after ligation release to study the process of regeneration. They found that expression of AQP5 increased after ligation release [41]. These findings in disagreement with our results and this may attributed to different treatment methods and our study was based on regeneration of parotid glands tissue not SMGs.

## Conclusion

Within the results obtained from this study, we suggest that MET, when loaded on CNPs, revealed better regenerative capacity on SGs healing than when used as a free drug. As it showed enhanced wound healing with increased function, as shown by increased AQP5 expression and decreased fibrosis. However, some limitations should be stated, as considering extending the observation period to 2–3 months to assess long-term healing and using barrier membranes to evaluate its effect in conjunction with MET-CNPs.

## Supplementary Information

Supplementary Material 1

## Data Availability

The data used during the current study are available from the authors on a reasonable request.

## Abbreviations

ANOVA: Analysis of Variance
AOD: After operation day
AP: Apple pectin
AQP5: Aquaporin 5
ARRIVE: Animal Research: Reporting of *In Vivo* Experiments
BM: Basement membrane
bFGF: Basic fibroblast growth factor
DDs: Drug delivery systems
EE%: Percent entrapment efficiency
FDA: Food and Drug Administration
FGF7: Fibroblast growth factor 7
GRAS: Generally Recognized as Safe
H&E: Hematoxylin and eosin
IHC: Immunohistochemical
MERC: Mansoura Experimental Research Center
MET: Melatonin
MET-CNPs: Melatonin loaded chitosan nanoparticles
NPs: Nanoparticles
P CNPs: Plain chitosan nanoparticles
PBS: Phosphate buffer saline
PDI: Polydispersity index
ROS: Reactive oxygen species
RT-PCR: Real time polymerase chain reaction
SD: Standard deviation
SGs: Salivary glands
SGTs: Salivary gland tumors
SMGs: Submandibular glands
Sodium CMC: Sodium carboxymethylcellulose
TEM: Transmission electron microscopy
TPP: Sodium tripolyphosphate
UPW: Ultrapure water
UV–VIS: Ultraviolet/visible
Z-average: Average particle size
ζ-potential: Zeta potential
