# Supplementary figures and images for: Melatonin free form versus its chitosan-loaded nano formula impact on wound healing of albino rat’s parotid gland “a histological study”
Source: BMC Oral Health. 2025 Oct 21;25:1653. doi: 10.1186/s12903-025-06869-4 (PMC12542121; doi:10.1186/s12903-025-06869-4)

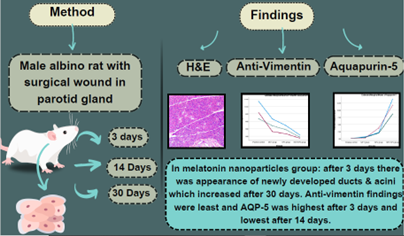

Supplement: Supplementary file 1 — Supplementary Material 1 [file 12903_2025_6869_MOESM1_ESM.png]
